# Supplementary material for: Understanding the relationship between apathy, cognition and functional outcome in schizophrenia: The significance of an ecological assessment
Source: PLoS One. 2022 Nov 3;17(11):e0277047. doi: 10.1371/journal.pone.0277047 (PMC9632867; doi:10.1371/journal.pone.0277047)
Supplement: S1 Table — (PDF) [file pone.0277047.s001.pdf]

S1 Table. Details of the measures used for cognition, negative symptoms, depression and functionality.

Symptom	Scale	Sub-Scales
Cognition	Executive Functions	Brief Assessment of Cognition in Schizophrenia
		Symbol Coding (BACS-SC): number of correct responses
		Trail Making Test- A: total time to complete the task
		Trail Making Test B: total time to complete the task
		Verbal Fluency: number of correct responses
		Composite score of executive functions (CEF)
	Executive Functions (ecological evaluation)	Jansari Assessment for Executive Functions:
		- Planning
		- Prioritization
		- Selection
		- Creative thinking
		- Adaptive thinking
		- Action-based prospective memory
		- Event-based prospective memory
		- Time-based prospective memory
		- Overall score
Apathy	Apathy Evaluation Scale (AES-C) - Clinical Version: total score	
Functionality	Functioning Assessment Short Test:	
	- Autonomy	
	- Occupational functioning	
	- Cognitive functioning	
	- Financial issues	
	- Interpersonal relationships	

	- Leisure time	
	- Overall Score	
Control measures	Negative Symptoms	Positive and Negative Syndrome Scale:
		- Positive
		- Negative
		- Cognitive / Disorganization
		- Excitability / Hostility
		- Depression / Anxiety
		- Total Score
		Brief Negative Symptom Scale: Total Score
		- Anhedonia
		- Distress
		- Asociality
		- Avolition
		- Blunted affect
		- Alogia
		- Total Score
	Depression	CDS: total score
		Mini- International Neuropsychiatric Interview – modules A, B and C
	Substance Abuse	Mini- International Neuropsychiatric Interview: modules J and K
	Vocabulary	Vocabulary sub-test from the WAIS-III
