# Supplementary material for: Understanding the relationship between apathy, cognition and functional outcome in schizophrenia: The significance of an ecological assessment
Source: PLoS One. 2022 Nov 3;17(11):e0277047. doi: 10.1371/journal.pone.0277047 (PMC9632867; doi:10.1371/journal.pone.0277047)
Supplement: S2 Table — (PDF) [file pone.0277047.s002.pdf]

S2 Table. Correlations between cognition, apathy, functionality, clinical variables, and sociodemographic characteristics.

	1	2	3	4	5	6	7	8	9	10
1. AES-C	1.000									
2. JEF	-.546*	1.000								
3. CEF	-.380	.457*	1.000							
4. FAST	.817**	-.567**	-.474*	1.000						
5. BNSS	.680**	-.636**	-.278	.503*	1.000					
6. CDS	.083	-.088	.008	.287	.037	1.000				
7. PANSS	.423	-.698**	-.440	.506*	.565**	.159	1.000			
8. Age in years	-.172	-.160	-.415	.128	-.456*	-.049	.172	1.000		
9. Years of education	-.661**	.683**	.410	-.624**	-.800**	-.298	-.606**	.217	1.000	
10. Age of onset of illness	-.158	.030	.097	-.161	-.228	.093	-.042	.329	.268	1.000

AES-C=Apathy Evaluation Scale - Clinical version; JEF=Jansari Assessment of Executive Functions; CEF=Composite score of executive function assessment; FAST=Functionality Assessment Short Test; BNSS= Brief Negative Symptom Scale; CDS= Calgary Depression Scale; PANSS=Positive and Negative Syndrome Scale.

* significance level at .05, ** significance level at .01.
