# Supplementary material for: Understanding the relationship between apathy, cognition and functional outcome in schizophrenia: The significance of an ecological assessment
Source: PLoS One. 2022 Nov 3;17(11):e0277047. doi: 10.1371/journal.pone.0277047 (PMC9632867; doi:10.1371/journal.pone.0277047)
Supplement: Attachment — Submitted filename: Review PONE-D-21-39885.docx [file pone.0277047.s003.docx]

The current study examined how an ecological cognitive assessment (the Jansari Executive Function Assessment, JEF) is associated with other variables as between cognition, apathy, and functional status in a sample of patients with schizophrenia. Using The Apathy Evaluation Scale (AES), a neuropsychological battery and the JEF, authors assessed 20 patients with schizophrenia. Data were analysed using a hierarchical multiple regression methodology. Finally, mediation analysis was performed to test the associations between the variables of interest. Authors reported a significant portion of the variance in AES (25%) being explained by the performance in JEF. Additionally, apathy explained 36% of the variance in functional outcome. Unfortunately, AES did not mediate between cognition and functional outcome. Generally speaking, is a well written and interesting paper although some aspects could be in need of some deeper analyses. Otherwise, it could be difficult to interpret some conclusions of the study.

- Do the authors consider that traditional neuropsychological tests included in the study are mirroring the same executive function subcomponents measured by the JEF? How can we interpret results if they are not measuring the same subcomponents?
- Another important issue is the analysis of the potential confounders. Some aspects as medication dose, type of antipsychotic, combination with other medications, sociodemographic characteristics could have an effect on cognition and affective variables. Could the authors try to incorporate the analyses of those confounders in their analyses? If not possible, authors could discuss their potential influence in the discussion as a limitation of the study.
- Authors use multiple regression and correlation coefficients and performed many statistical analyses using a relatively small sample. How the authors determined the sample size? Did they perform any correction for multiple comparisons?
